# Task allocation in a cooperative society: specialized castes or age-dependent switching among ant workers

**DOI:** 10.1038/s41598-020-59920-5

**Published:** 2020-02-24

**Authors:** Yoh Iwasa, Sachi Yamaguchi

**Affiliations:** 1 Department of Bioscience, School of Science and Technology, Kwansei Gakuin University, Gakuen 2-1, Sanda, 669-1337 Japan; 2 KYOUSEI Science Center for Life and Nature, Nara Women’s University, Kitauoyahigashi-machi, Nara, 630-8506 Japan

**Keywords:** Behavioural ecology, Evolutionary ecology, Applied mathematics

## Abstract

Some ant species have multiple worker castes that differ in body size; workers in one caste remain in the colony and those in the other forage outside the colony (caste polyethism). In other species, all workers engage in both tasks, but the younger workers remain in the colony and the older workers forage (age polyethism). Here, we ask which of these two is the most efficient for colony level performance when foragers suffer a higher daily mortality than workers in the colony and when the optimal worker size differs between two tasks. We studied two models: in the stationary colony model, the colony size and composition remain constant, and the amount of excess resources that can be used for producing reproductive individuals is maximized; in the growing colony model, all of the resources obtained are used for producing new workers, and the rate of the colony growth is maximized. In both models, we observed similar results: caste polyethism is more advantageous than age polyethism if the difference in mortality between the two tasks is small and the difference in the optimal size is large. In the opposite situation, the age polyethism is more advantageous.

## Introduction

Workers of social insects, such as ants, perform many different tasks, including caring for eggs and larvae; cleaning and patrolling the colony; foraging; and defending against invaders, parasites, and predators.

In some species, workers have several morphologically distinct types called “castes,” which tend to be specialized for different tasks^1^. For example, in weaver ants of the genus *Oecophylla*, one caste of smaller body size (minor workers) is specialized for in-colony work, while the second caste of larger body size (major workers) is specialized for foraging, nest construction, or other out-of-colony work^2^. In the genus *Pheidole*, some species have two castes, but others have more^3–5^. In leaf-cutting ants in the genus *Atta*, there are workers in multiple castes with different size and morphology, performing different tasks^6^.

By contrast, individuals in many other species engage in different tasks based on their age (time since emergence). Typically, younger workers remain in the colony to take care of eggs and larvae, and older workers forage outside the colony^7^. This phenomenon, termed “age polyethism”^1^, is widely observed in ants, as well as in other social insects including honey bees. Performing the task of a higher daily mortality in older ages is better than other age-dependent patterns of allocating tasks in computer simulations^8^.

Many papers have been published regarding the task allocation in ant colonies, focusing on how the simple mechanistic reaction of each individual can generate a complex adaptive behavior within the whole colony. For example, Bonabeau *et al*.^9^ and Theraulaz *et al*.^10^ studied individual-based models in which each worker switches its behavior based on signals indicating the needs of different tasks. Each individual worker has its own quantity based on its experiences, and when the quantity passed a value (called threshold), then the individual changes the behavior. The models exhibited the flexible and adaptive allocation of workers to tasks similar to the patterns observed among ant colonies. Duarte *et al*.^11^ examined the adaptive significance of a behavior by the entire colony by adding a natural selection process to self-organized threshold models. Duarte *et al*.^11,12^ also discussed a condition in which each worker specializes in each task or remains a generalist that responds to different tasks.

In this paper, we study the condition in which caste polyethism or age polyethism is more efficient than the other in terms of colony performance. We investigate two different models handling different phases of colony growth.

In the first model, we consider a stationary colony in which the colony size and the composition do not change with time. The colony produces new workers to maintain the colony and replenish the mortality loss of workers; however, it also produces reproductive individuals (males and new queens) out of the resources obtained by the workers, minus the investment for replenishing workers. Natural selection would produce the polyethism pattern that achieves the largest excess of resources that can be used for producing reproductive individuals.

To consider a stationary colony, we set the following assumptions: the amount of resources usable for new worker production is a given constant; the number and size of workers can be chosen by the colony as their strategy under this resource constraint; workers have a fixed maximum age ($${a}_{max})$$; workers engage in two different tasks (working in the colony [taking care of eggs and larvae and cleaning the colony] and foraging outside the colony); as foraging is accompanied by danger caused by predators, parasites, and physical accidents, foragers suffer a higher daily mortality than workers in the colony.

We first analyze a case in which two castes existed: one that specializes in the work in the colony and one that specializes in foraging (caste polyethism). They may differ in body size. The number of new workers and the body sizes of the two castes can be chosen to maximize the reproductive output of the colony, given the limitation of the total amount of resources usable for worker production per day. Next, we study a case in which all the workers are of the same size and perform both tasks according to their age: younger workers remain in the colony, while older workers engage in foraging (age polyethism). Here the body size and the age for switching between the two tasks are chosen optimally. Finally, we compare the colony of workers of the same size performing both tasks (age polyethism) and the colony of workers specialized for different tasks (caste polyethism); we discuss the condition in which one is more productive than the other.

In contrast, in the second model, we consider a growing colony. The colony is in an early phase of colony growth, where no new reproductive individuals (males and new queens) appear, and continues to grow by producing new workers using all of the resources obtained by their labor. The colony will produce reproductive individuals much later when the colony size reaches sufficiently large numbers. In the growing colony model, we measure the performance of the colony by their growth rate. We consider a situation in which the work efficiency of each worker and their mortality may depend on their body size or their activities, but are independent of the colony size. From this assumption, the colony will begin to grow exponentially; we are able to use the exponential rate of colony growth as the criterion to evaluate the performance of different polyethism patterns.

## Models and Results

### Stationary colony model

Here, we study the stationary colony model, which is easier to analyze mathematically than the growing colony model. We consider the simplest possible model in which the colony is in a stationary state, with a constant age structure of workers.

#### Body size and number of specialized workers

The ant workers are adults, and their body sizes do not change once they emerge from the pupal stage. Their body size is determined by the amount of resources supplied in the growing period. Two phenotypes exist: workers specialized for work in the colony (body size ${x}_{1}$ and number produced per day ${n}_{1}$) and workers specialized for foraging outside the colony (body size ${x}_{2}$ and number produced per day ${n}_{2}$). Note that ${n}_{1}$ and ${n}_{2}$ are the daily influx of workers, and they replenish the loss of workers due to daily mortality to maintain the same composition. For simplicity, we assume that the cost of producing a worker is proportional to the body size. In this paper, we term ${x}_{1}$ and ${x}_{2}$ as the body size of workers in the two castes, but to be more accurate, they indicate the costs for producing workers in the two different castes.

**Figure 1:**
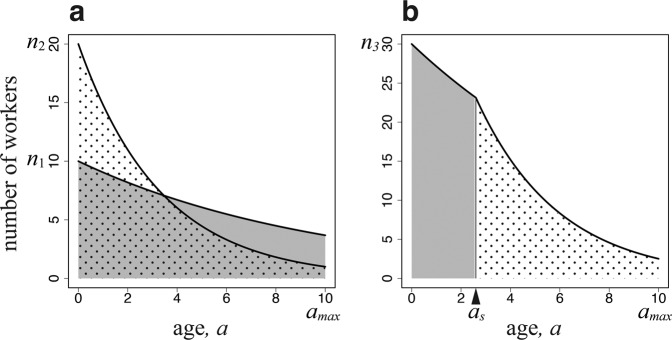
Number of workers engaging in two tasks as a function of age. The horizontal axis is $a$, i.e., the time since emergence (age polyethism). (**a**) There are two groups of workers, each specialized in one task (caste polyethism). (**b**) There is a single type of worker engaging in both tasks according to age. The parameters used are as follows: $u=0.1$, $v=0.2$, ${a}_{max}=10$; (**a**) ${n}_{1}=10$, and ${n}_{2}=20$; (**b**) ${n}_{3}=30$, and ${a}_{s}=2.6$.

**Figure 2:**
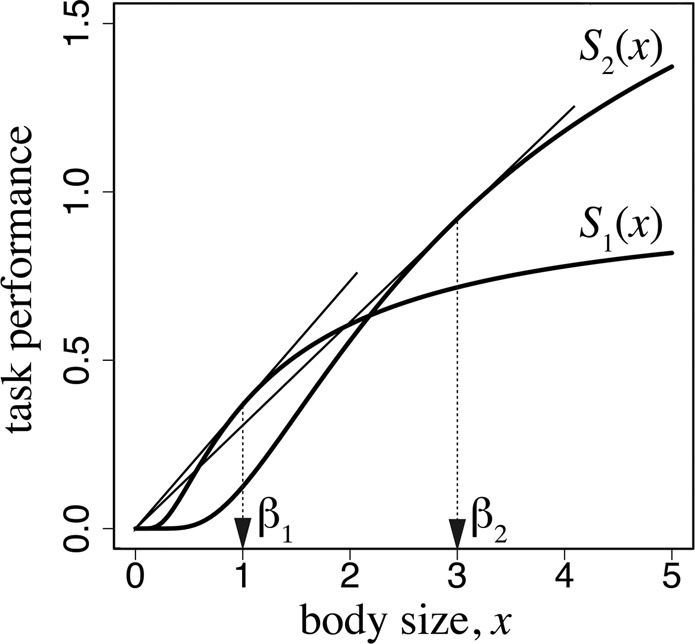
Task performance as a function of worker body size. The parameters used are as follows: ${\alpha }_{1}=1.0$, ${\beta }_{1}=1.0$, ${\alpha }_{2}=2.5$, and ${\beta }_{1}=3.0$.

The total amount of work within a colony is given by the summed number of workers of different ages, which decreases with age owing to mortality (Fig. 1a). The workers specialized for the work in the colony suffer daily mortality $u$, and their age structure is ${n}_{1}\exp [-ua]$ for $0 < a < {a}_{max}$. Each day, a surviving worker of this type performs a task of amount ${S}_{1}({x}_{1})$. Here, we assume that it is a sigmoid function of body size ${x}_{1}$, which is an assumption commonly adopted when discussing optimal egg size^13^. This indicates that a very small worker cannot perform well; however, beyond a certain size, the performance rapidly increases with body size, followed by performance saturation for large sizes (See Fig. 2). Hence, the total amount of tasks performed by these specialized workers in the colony is as follows:

1a $$I={\int }_{0}^{{a}_{max}}\,{n}_{1}\exp [-ua]{S}_{1}({x}_{1})da={n}_{1}{S}_{1}({x}_{1}){C}_{1},$$

where ${C}_{1}=\,\frac{1}{u}(1-\exp [-u{a}_{max}])$. ${C}_{1}$ implies the effective number of days for a newly emerged worker to contribute to the colony until its death (by either daily mortality or maximum age).

Similarly, the age structure of workers specialized for foraging is ${n}_{2}\exp [-(u+v)a]$ for $0 < a < {a}_{max}$. Foraging is accompanied by a higher daily mortality than is working in the colony, and we denote the excess mortality by $v$. Let ${S}_{2}({x}_{2})$ be the foraging performance per day by a surviving worker. The total foraging performance of the entire colony per day is the product of the per capita foraging performance ${S}_{2}({x}_{2})$ and the number of workers of age $a$ integrated from 0 to ${a}_{max}$, and is given as follows:

1b $$F={n}_{2}{S}_{2}({x}_{2}){C}_{2},$$

where

$${C}_{2}=\frac{1}{u+v}(1-\exp [-(u+v){a}_{max}]).$$

Here, we discuss the performance of producing ants of the whole colony. This requires both the work within the colony and the work out of the colony. The abundance of one task (say working within the colony) cannot compensate for the shortage of the other task (foraging) and vice versa. The performance of the entire colony should be limited by the performance of the most limited task between the two (Liebig’s law of the minimum^14,15^). We denote the work performance in the colony by $I$ and that of foraging by $F$. Additionally, we adopt the following simple assumption:

2 $$B=\,{\rm{\min }}[I,F].$$

Note that, by scaling task performance functions ${S}_{1}(x)$ and ${S}_{2}(x)$ appropriately, we can always make the most efficient use of labor is achieved when the total performance of two tasks are equal: $\,I=F$. Wakano *et al*.^8^ adopted equation (2) for the performance of the colony. They studied the situation in which $I$ and $F$ are multiplied by stochastically changing coefficients, because they are considering the effect of environmental fluctuation, which we do not consider in this paper. In the equation above, $B$ is the amount of resources for producing new workers and new reproductive individuals (such as new queens and males). Let $R$ be the fixed amount of resources to be used for producing new workers per day. Because the success of the colony is the production of reproductive individuals, we can use $B-R$ as a criterion to measure the performance of the entire colony. Here, we treat $R$ as a fixed constant, and we regard $B$ as the quantity to maximize in the following argument.

We search for a combination of ${n}_{1}$, ${x}_{1}$, ${n}_{2}$, and ${x}_{2}$ that achieves the maximum $B$ in equation (2) under the following resource constraint:

3 $$R={n}_{1}{x}_{1}+{n}_{2}{x}_{2},$$

where $R$ is a constant given as a parameter of the model.

In SI Appendix A, we provide the analysis of this optimization problem. The results show that the optimal sizes of the two workers ${x}_{1}$ and ${x}_{2}$ can be obtained from the following formulas:

4a $${S}_{1}({x}_{1})/{x}_{1}={S}_{1}\text{'}({x}_{1}),$$

4b $${S}_{2}({x}_{2})/{x}_{2}={S}_{2}\text{'}({x}_{2}).$$

These equations are the same as the optimal solutions in the model of the optimal number and size of eggs^13^. As illustrated in Fig. 2, the optimal body size ${x}_{1}$ is the value of the tangential point obtained from drawing a line passing through the origin to the curve of function ${S}_{1}({x}_{1})$. Similarly, the optimal size of foraging workers ${x}_{2}$ can be obtained by drawing a line passing through the origin to the curve of function ${S}_{2}({x}_{2})$. This implies that the body size of each caste is determined by the graph of the task performance as a function of body size for each task. Different tasks may require different body sizes, as represented by the difference between ${S}_{1}(x)$ and ${S}_{2}(x)$. Consider the case in which a worker of a smaller body size is sufficiently efficient in work within the colony; therefore, the optimal size for the caste specialized for working inside the colony is small (in terms of the cost for producing one worker). By contrast, if efficient foraging requires a larger worker, the optimal size for the foraging ant caste becomes larger.

In Fig. 2, we adopted ${S}_{i}(x)={\alpha }_{i}\exp [-\frac{{\beta }_{i}}{x}]$ for $i=1,\,2$. For these functions, the optimal sizes in equation (4) are simply ${x}_{i}={\beta }_{i}$ ($i=1,\,2$).

The numbers of workers produced per day for the two castes are determined as follows:

5a $${n}_{1}=\frac{R}{{x}_{1}}\frac{\frac{{x}_{1}}{{S}_{1}({x}_{1}){C}_{1}}}{\frac{{x}_{1}}{{S}_{1}({x}_{1}){C}_{1}}+\frac{{x}_{2}}{{S}_{2}({x}_{2}){C}_{2}}},$$

5b $${n}_{2}=\frac{R}{{x}_{2}}\frac{\frac{{x}_{2}}{{S}_{2}({x}_{2}){C}_{2}}}{\frac{{x}_{1}}{{S}_{1}({x}_{1}){C}_{1}}+\frac{{x}_{2}}{{S}_{2}({x}_{2}){C}_{2}}}.$$

The derivation of these results is provided in SI Appendix A.

The amount of resources used for the small workers is ${n}_{1}{x}_{1}$ per day and that for the large workers is ${n}_{2}{x}_{2}$ per day. Equations (5a) and (5b) indicate that the amount of resources available for new worker production per day, $R$, is split between the small workers and large workers at a ratio of $\frac{{x}_{1}}{{S}_{1}({x}_{1}){C}_{1}}$ to $\frac{{x}_{2}}{{S}_{2}({x}_{2}){C}_{2}}$. It is noteworthy that ${S}_{i}({x}_{i})/{x}_{i}$ can be interpreted as the efficiency of investment in the production of workers of type $i$ measured by the daily amount of task $i$ to be performed per unit resource investment. ${C}_{i}$ is the expected number of days that a worker lives. Hence, equation (5) implies that the amount of resources to be used in the optimal colony strategy is to be invested in a manner inversely proportional to the task-performing efficiency. More resources should be allocated to the task with the lower efficiency. For example, in a habitat with abundant food availability, a smaller fraction of resources should be allocated to forager production than in an environment with scarce food resources.

#### When workers perform both tasks in an age-dependent manner

We now consider a colony that consists of workers of the same size ${x}_{3}$, which performs both tasks depending on age. As illustrated in Fig. 1b, they engage in work in the colony before switching age ${a}_{s}$ and in foraging outside the colony after ${a}_{s}$. The shaded and dotted areas are individuals working in the colony and foraging outside of the colony, respectively. Foraging is accompanied by a higher daily mortality than working in the colony. Let ${n}_{3}$ be the number of newly emerging workers per day. If they switched tasks based on age, the younger workers would engage in tasks with smaller daily mortality, while the older workers would perform tasks with larger daily mortality. This is optimal for the entire colony. Because the older workers have a shorter time remaining until the maximum age ${a}_{max}$ than the younger workers, the risk of higher mortality would be less important than in the younger workers, as the latter have more days before reaching ${a}_{max}$. This pattern has been observed in many species^1,7^, and was confirmed by computer simulations of colony growth^8^. Therefore, we assume this pattern in our study. We chose the switching age ${a}_{s}$ to be the optimal age to attain the maximum colony performance. Furthermore, we optimized the size ${x}_{3}$ and the number of workers ${n}_{3}$ to be produced per day. The total amount of work within the colony and that of foraging are as follows:

6a $$I={\int }_{0}^{{a}_{s}}\,{n}_{3}\exp [-ua]{S}_{1}({x}_{3})da={n}_{3}{S}_{1}({x}_{3}){A}_{1}({a}_{s}),$$

6b $$F={\int }_{{a}_{s}}^{{a}_{max}}\,{n}_{3}\exp [-u{a}_{s}]\exp [-(u+v)(a-{a}_{s})]{S}_{2}({x}_{3})da={n}_{3}{S}_{2}({x}_{3}){A}_{2}({a}_{s})$$

respectively. Here, we define two quantities:

7a $${A}_{1}({a}_{s})=\frac{1}{u}(1-\exp [-u{a}_{s}]),$$

7b $${A}_{2}({a}_{s})=\exp [-u{a}_{s}]\frac{1}{u+v}(1-\exp [-(u+v)({a}_{max}-{a}_{s})]).$$

Each implies the effective number of days to contribute to the colony through the respective task to be made by a newly emerged worker. The objective function is, again, the colony performance given by equation (2), which is maximized under the resource constraint: $R={n}_{3}{x}_{3}$.

According to the analysis in SI Appendix B, the optimal solutions of ${a}_{s}$ and ${x}_{3}$ satisfy the following:

8 $$\frac{\frac{d}{d{a}_{s}}\,\mathrm{ln}\,{A}_{1}({a}_{s})}{\frac{1}{{x}_{3}}\,-\frac{d}{\,d{x}_{3}}\,\mathrm{ln}\,{S}_{1}({x}_{3})}=\frac{\frac{d}{d{a}_{s}}\,\mathrm{ln}\,{A}_{2}({a}_{s})}{\frac{1}{{x}_{3}}\,-\frac{d}{\,d{x}_{3}}\,\mathrm{ln}\,{S}_{2}({x}_{3})}.$$

We also have ${S}_{1}({x}_{3}){A}_{1}({a}_{s})={S}_{2}({x}_{3}){A}_{2}({a}_{s})$, indicating that the total amount of work within the colony and that of foraging outside the colony must be balanced in the optimal use of labor ($I=F$). Using these two equations, we can solve the optimal solution numerically (i.e., the age of switching ${a}_{s}$ and the size ${x}_{3}$). The optimal number of eggs is then calculated as ${n}_{3}=R/{x}_{3}$.

To determine the behavior of the model, we specify a task-performance function as follows: ${S}_{i}(x)={\alpha }_{i}\exp [-{\beta }_{i}/x],$ with $i=1,2$. According to the analysis in SI Appendix B, we can determine the optimal worker size ${x}_{3}$ and the optimal age of switching ${a}_{s}$ which satisfy equations (7) and (8), using these particular forms of task performance functions. When workers engage in both tasks in an age-dependent manner, the optimal worker size ${x}_{3}$ lies between ${\beta }_{1}$ and ${\beta }_{2}$, which are the optimal sizes for specialized workers (see equations (B.7a) and (B.7b)).

#### Competition between specialized castes and age-dependent switching

We now consider the competition between a type with caste polyethism and another type with age polyethism. The worker sizes and numbers are chosen to maximize the colony performance, as described in the previous two sections.

**Figure 3:**
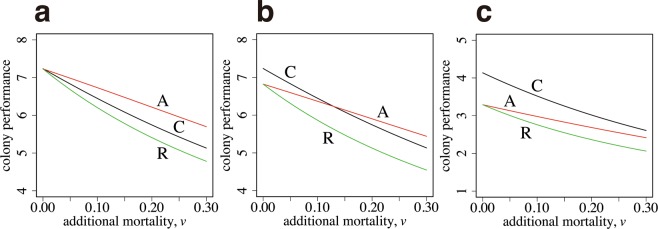
Colony performance of age polyethism and caste polyethism. (**a**) The task-performance function is the same between the two tasks (${\alpha }_{1}={\alpha }_{2}=1.0,$ ${\beta }_{1}={\beta }_{2}=1.0$). (**b**) The optimal size for foraging is larger than that for the in-colony work (${\beta }_{1}=1.0$, ${\beta }_{2}=2.0$). The parameters in (**b**) are as follows: ${\alpha }_{1}=1.0$, and ${\alpha }_{2}=2.0$. (**c**) The optimal sizes differ more (${\beta }_{1}=1.0$, ${\beta }_{2}=5.0$). The parameters in (**c**) are as follows: ${\alpha }_{1}=1.0$, and ${\alpha }_{2}=2.0$. The other parameters used are as follows: ${a}_{max}=5.0$, $u=0.1$, and $R=10$.

We start with the case in which the performance–size relationships for two tasks, ${S}_{1}(x)$ and ${S}_{2}(x)$, are equal. Therefore, the optimal body size is the same for both tasks. A curve, labeled as A in Fig. 3a, illustrates the colony output of the case with age polyethism, with the number of new workers ${n}_{3}$ and switching age ${a}_{s}$ chosen as the optimal values, given the constraint $I=F$, the latter being from the need that $I$ and $F$ must be equal to realize the optimal. The curve labeled as C is the result of optimal caste polyethism, with ${n}_{1}$ and ${n}_{2}$ chosen to maximize the colony performance $B$, given by equation (2). The horizontal axis is $v$, which represents the additional daily mortality for foraging. The mortality of workers working within the colony is fixed as $u=0.1$. As $v$ increases, the colony performance declines but that of age polyethism is always greater than that of caste polyethism. We also calculated a curve, labeled as R, which indicates the performance of “reverse age polyethism”, in which foraging (the activity with a higher mortality) is first performed and then ants switch to work in the colony (the activity with a lower mortality) later in life. The performance is always worse than that of age polyethism with the correct order. Note that if we multiply the same factor to all the rate constants in the model, the qualitative behavior of the model remains the same. What controls the relative advantage of different polyethism in Fig. 3 is the ratio between mortalities $v/u$.

Figure 3b illustrates the case in which the optimal body size differs between two tasks. Two curves, labeled as C and A, cross each other. For a small $v$, caste polyethism generates a greater performance than age polyethism. However, for a large $v$, age polyethism performs better than caste polyethism.

Figure 3c illustrates the case in which the optimal body size differs significantly between two tasks; the optimal size for foraging is five times larger than that for working within the colony. Caste polyethism performs better than age polyethism for all values of $v$ shown in the figure.

**Figure 4:**
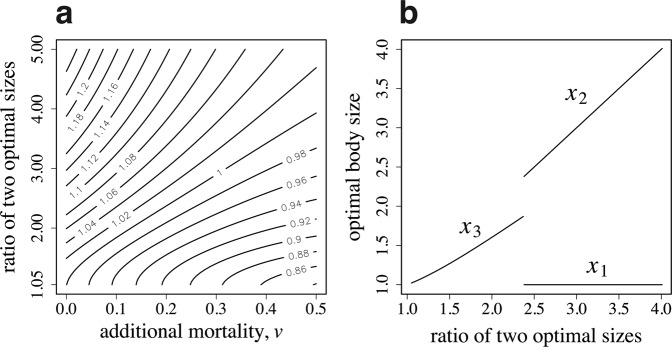
Age polyethism versus caste polyethism. (**a**) Contour map of the relative advantage of specialized castes to age-dependent task switching. Numbers by the contours indicate the ratio of colony performance $B$ of caste polyethism to age polyethism. (**b**) The optimal size of workers to produce. The parameter used in (**b**) is $v=0.2$. The other parameters are as follows: ${\beta }_{1}=1.0$, ${\alpha }_{1}=1.0$, ${\alpha }_{2}=2.0$, ${a}_{max}=5.0$, $u=0.1$, and $R=10$.

Figure 4a illustrates the relative success of the caste specialization over age-dependent task switching in a contour map. The horizontal and vertical axes represent the additional mortality due to foraging $v$ and the ratio of two optimal sizes ${\beta }_{2}/{\beta }_{1}$, respectively. The contour map shows that caste polyethism is more advantageous when ${\beta }_{1}$ and ${\beta }_{2}$ differ more strongly and when the additional mortality $v$ is small; while age polyethism is more advantageous in the opposite situation. Figure 4b illustrates the optimal worker sizes. The horizontal axis represents the ratio of two optimal sizes. If ${\beta }_{2}/{\beta }_{1}$ is small, all workers are of the same size ${x}_{3}$; however, if ${\beta }_{2}/{\beta }_{1}$ is large, dimorphic workers are produced with sizes ${x}_{1}$ and ${x}_{2}$. We can see the transition from single-sized workers (with age-dependent switching of tasks) to the two castes differing in body size.

#### Effect of maximum worker age

**Figure 5:**
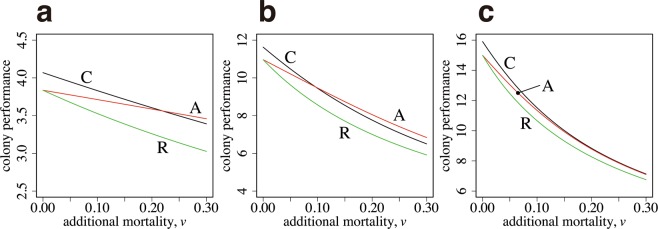
Effect of maximum worker age. This figure is similar to Fig. 3. The maximum worker ages are as follows: (**a**) ${a}_{max}=2.5$, (**b**) ${a}_{max}=10$, and (**c**) ${a}_{max}=20$. Other parameters are the same as those in Fig. 3b.

The advantage of age polyethism (i.e., performing foraging after the work in the colony) is caused by the existence of the maximum age of workers. This effect diminishes as the maximum age becomes larger. Figure 5 illustrates that, when the maximum age of the workers is very large and only a negligible portion of the workers can reach the maximum age, performing foraging after working in the colony becomes similar in terms of colony performance to performing foraging before working in the colony.

### Growing colony model

We previously focused on the situation of a stationary population. However, in the early stage of colony development, we should consider the optimal polyethism in the colonies that grow exponentially with time. In these colonies, all of the resources obtained should be allocated to produce new workers instead of reproductive individuals. We assume that the production of new workers is limited by the performance of labor by current workers, but not by the egg production capacity of queen(s). Then we replace equation (2) with $R=\,{\rm{\min }}[I,F]$, which implies that the amount of resources usable for new worker production is equal to the resources obtained by the colony per day. All of the variables become functions of time $t$.

We here assume that the model does not contain density-dependent processes, Then, starting from any initial distribution, the population eventually begins to grow exponentially, in which all of the age classes increase by a factor of ${e}^{rt}$, in which $r$ is the Malthusian parameter, or the dominant eigenvalue in a dynamics model. The rate of exponential growth is the criterion for colony performance. We search for the optimal task allocation strategy that achieves the maximum value of $r$. This logic was proven by Taylor *et al*.^16^ and Leon^17^ in the context of life history evolution. We provide an analysis for the optimal polyethism in SI Appendix C.

Quantitatively, the results are the same as those of the model for stationary populations explained in earlier sections. For example, if the colony consists of two castes specialized for working in the colony and foraging outside the colony, the worker sizes should evolve according to equations (4a) and (4b): exactly the same sizes as those for the stationary colony. Next, the ratio of resource amounts to be allocated to the two castes is inversely proportional to the task-performing efficiency per unit investment; however, the quantities *C*_1_ and *C*_2_ representing the effective number of days to survive include the time-discounting effect with rate *r* (see SI Appendix C for details). Equations similar to equations (5a) and (5b) hold, but $R$ must be replaced by the supply of resources available for the production of workers, and the ratio must be modified considering the time-discounting factor—a task performed in the future is less effective than the same task performed in the present in an exponentially growing population. A similar modification occurs in a single phenotype with task switching based on age (see SI Appendix C).

## Discussion

Specializing in one task could improve the skill, and thus the task performance might be improved compared to that when combining two tasks. The role of behavioral specialization has been emphasized in papers on the adaptive significance of age polyethism versus caste polyethism^11,12^. Morphological difference should give an even clearer reason for the advantages of specialization compared to behavioral specialization. In this paper, we focus on the body size of the workers. Even if both tasks can be performed better by a worker with a large body, the relative advantage considering the resources needed to produce a new worker may differ between the two tasks, and the optimal size should be determined as that at the tangential points of the $S(x)$-graph and a line passing through the origin, or the one that maximizes $S(x)/x$. This may differ between tasks. For example, in a given species, foraging might require the optimal worker size to be larger than the within-colony worker size. However, for age-dependent task switching, the species is constrained to produce workers of a size that is the intermediate between the two optima, which is too small for a forager but too large for a within-colony worker. The advantage of caste polyethism is that the optimal body sizes for both tasks can be achieved. However, this cannot be achieved for workers of a single type that perform both tasks. They must choose one body size, thus compromising the requirements of the different tasks.

When multiple castes specializing in different tasks exist, the optimal ratio depends on the efficiency of the task performance. The optimal resource allocation to each caste is inversely proportional to the efficiency, implying that more foragers should be produced when the food availability in the environment is low than when it is high. However, the availability of food items is likely to fluctuate.

Here, we investigate the relative advantage of colonies containing workers of two different sizes and those containing workers of a single phenotype that perform both roles. Concerning the body size, the former is more efficient than the latter in the use of limited resources for worker production, because the workers engaging in each task have the optimal size for the respective task, while the latter must employ an intermediate body size that is too small for one task but too large for the other. In contrast, the latter is favored by the differential daily mortality of the two tasks, because performing the task with a higher daily mortality after the one with a lower mortality is more efficient. The relative advantage of the two is determined by the relative magnitude of the two processes. Interestingly, a very similar situation is observed concerning the larval size of a marine organism^18^. Parasitic (rhizocephalan) barnacles include species in which the larval sex is determined by the mother, as male larvae are larger than female larvae. Other species of parasitic barnacles have monomorphic larvae that can become either male or female depending on the condition of the host in which they settle (environmental sex determination). The former is advantageous because male and female larvae have sizes that are optimal for their respective tasks. In contrast, being able to adjust the sex after encountering a host is advantageous because each larva has a higher chance of encountering an acceptable host. The relative advantage of the two should be determined by the importance of the two processes^18^.

In this paper, we analyzed the most efficient polyethism both in a stationary colony and in a growing colony. In a stationary colony, the resource gain, minus the investment for maintaining the colony size, would be used for producing reproductive individuals, and hence we adopted the excess gain of resources as the criterion for the performance of different models of polyethism.

In contrast, in a growing colony, no reproductive individuals are produced. All of the resources gained from the labor of the workers are invested to produce new workers, without producing reproductive individuals. The size of the colony increases, and the rate of colony growth is adopted as a measure of the adaptiveness of different polyethisms. Since we considered the case in which the survivorship, task-performance, and other properties of workers are independent of the colony size, the colony would begin growing exponentially, with a constant proportion of workers within the colony. The situation is mathematically equivalent to the basic model of demographic dynamics. Then, we searched for the optimal polyethism that achieves the maximum rate of exponential growth of the worker population.

In a growing colony, we examined the case of two castes with different body size (caste polyethism), where we chose the body sizes and number produced per unit time of the two castes as the values that achieve the maximum rate of colony growth. We also examined the case of a single caste with workers switching their task based on age (age polyethism), where we chose the body size and task-switching age as those that attain the maximum rate of colony growth. Finally, we compared the performance of the optimal caste polyethism and the optimal age polyethism in terms of the colony growth rate. This analysis is explained in SI Appendix C.

The results of the adaptive polyethism model were almost the same for the growing colony and stationary colony, except for the time-discounting factor to be considered in the growing colony. This implies that, in a rapidly growing colony, the performance of a task at an older age contributes less to the colony growth rate than the same amount of work being performed at a younger age. The performance of a task at worker age $a$ should be discounted by a factor of ${e}^{-ra}$, where $r$ is the exponential rate of colony growth. This time-discounting effect is known in the theory of life history strategy^16,17^.

In the growing colony model, new nonreproductive individuals are produced from the resources obtained by the existing nonreproductive individuals, and hence a model in which they look like the offspring of existing workers is useful in analyzing their dynamics. However, biologically speaking, they are not offspring of workers, but offspring of the queen. We may consider a very similar situation when we discuss the population of leaves on a single individual plant. Since the photosynthesis of old leaves would result in the production of new leaves, we may regard the growth of an individual plant as the growth of a population of leaves in which the photosynthesis performed by old leaves would result in the production of new leaves, just like the resources obtained by current worker ants results in the production of new workers. Certainly, new leaves are not offspring of existing leaves. This formalism was adopted when the optimal defense effort for leaves (alkaloid concentration) depending on the leaf age was discussed by Iwasa *et al*.^19^, wherein the time-discounting factor is used in calculating the optimal strategy of the plant.

We studied the growing colony model by assuming the rate of producing new individual workers are purely limited by the labor performance of the current workers. In general, the colony growth may be constrained by other factors as well, such as the egg producing capacity of queen(s). This may be a subject of future theoretical study.

We have shown that the method of calculating the optimal polyethism is similar between exponentially growing small colonies and large stationary colonies. However, in general, the optimal task allocation pattern of small growing colonies may be different from the best polyethism of large stationary colony. If so, as a colony grows in size, the polyethism pattern might change.

One aspect that was not investigated in this study is environmental fluctuation. The need of workers for the different two tasks is likely to fluctuate. Wakano *et al*.^8^ discussed the effect of environmental fluctuation in the evolutionarily stable allocation of workers according to age; however, they assumed that the age distribution of workers between two tasks is fixed. However, the number of workers engaging in different tasks would change in response to the current need for workers for different tasks (H. Shimoji, personal communication). For example, if food availability in a foraging site becomes high, a small number of foragers may be able to collect a sufficient amount of food. Subsequently, some of the workers that engage in foraging in the standard situation might start helping the workers within the colony. When food availability is low, more foragers are needed to collect the amount of food needed for colony maintenance, and workers that are supposed to be in the colony might start foraging. This flexibility should be applicable to both age polyethism and caste polyethism. We conjecture that older workers would be more likely to forage than younger workers, and larger-sized workers would be more likely to forage than small-sized workers, which are aspects handled by age polyethism and caste polyethism, respectively. Incorporating the effect of the flexible adjustment of worker numbers to both tasks would be worthwhile in future theoretical studies. Each individual worker must choose a task based on the needs of the tasks in the colony as well as its own age and body size.

Oster and Wilson^3^ discussed the numbers of workers in different castes and reproductive individuals for ant colonies. They introduced a linear programming technique to combine the achievement of multiple tasks by the workers in the colony. Wilson^1^ discussed the problem of the number of castes in the colony and compared it with the number of cell types of multicellular organisms. Bonner^20^ reported that the number of cell types of multicellular organisms tends to increase with the total number of cells. Although this is a widely observed pattern, the reason that the number of cell types increases with the total number of cells is unclear; similarly, the reason ants with larger colonies tend to have more castes among workers is unclear. The model from the current study cannot answer this question because the advantage of having two separate castes versus a single morphological type engaging in two tasks is independent of the total colony size. This is an interesting theoretical biology theme in social insects.

## Supplementary information

Supplementary Appendix.

## Data Availability

Data sharing is not applicable to this article as no datasets were generated or analyzed during the current study.
