# Supplementary material for: Task allocation in a cooperative society: specialized castes or age-dependent switching among ant workers
Source: Sci Rep. 2020 Feb 24;10:3339. doi: 10.1038/s41598-020-59920-5 (PMC7039887; doi:10.1038/s41598-020-59920-5)
Supplement: Supplementary file 1 — Supplementary Appendix. [file 41598_2020_59920_MOESM1_ESM.pdf]

SUPPLEMENTARY INFORMATION

(Appendices A-C)

Task allocation in a cooperative society: specialized castes or age-dependent switching among ant workers

Yoh Iwasa and Sachi Yamaguchi

Supplementary Information

Appendix A

Workers with two specialized castes

The model is explained in the text. Here, we consider a colony with workers of two different sizes, x_1 and x_2. They are produced (more exactly, emerge from pupae) at rates n_1 and n_2 per day, respectively. We search for the optimal combination of x_1, x_2, n_1, and n_2 that achieves the maximum value of equation (2) under the constraint of equation (3).

The criterion equation (2) can be rewritten as follows:

$$\phi = \min[n_1 S_1(x_1) C_1, n_2 S_2(x_2) C_2] \rightarrow \text{maximum} \qquad \text{(A.1)}$$

under the constraint of equation (3).

Since the criterion is achieved when two quantities are equal, we have

$$n_1 S_1(x_1) C_1 = n_2 S_2(x_2) C_2, \qquad \text{(A.2)}$$

in the optimal solution. Equation (A.2) can be treated as the second constraint. Hence, a problem is formulated in the search for the optimal solution, in which the maximum of ϕ:

$$\phi = n_1 S_1(x_1) C_1 \rightarrow \text{maximum}, \tag{A.3}$$

is achieved under two constraints: equation (A.2) and equation (3).

Using two Lagrange multipliers, λ and μ, the problem can be solved by considering the maximization of Φ, defined as,

$$\Phi = n_1 S_1(x_1) C_1 + \lambda\{n_1 S_1(x_1) C_1 - n_2 S_2(x_2) C_2\} + \mu\{R - n_1 x_1 - n_2 x_2\}. \tag{A.4}$$

Then, we calculate: $0 = \partial\Phi/\partial n_1$, $0 = \partial\Phi/\partial x_1$, $0 = \partial\Phi/\partial n_2$, and $0 = \partial\Phi/\partial x_2$, which leads to the following:

$$0 = (1 + \lambda) S_1(x_1) C_1 - \mu x_1, \tag{A.5a}$$

$$0 = (1 + \lambda) S_1'(x_1) C_1 - \mu, \tag{A.5b}$$

$$0 = -\lambda S_2(x_2) C_2 - \mu x_2, \tag{A.5c}$$

$$0 = -\lambda S_2'(x_2) C_2 - \mu. \tag{A.5d}$$

From equations (A.5a) and (A.5b), we can derive

$$S_1(x_1)/x_1 = S_1'(x_1), \tag{A.6a}$$

which is the optimal solution to the problem of searching for the number and size of offspring under the resource constraint (Smith and Fretwell 1974). In a similar manner, from equatiosn (A.5c) and (A.5d), we have the following:

$$S_2(x_2)/x_2 = S_2'(x_2). \tag{A.6b}$$

The optimal size x_1 is the value at the point of contact when we draw a tangential line passing through the origin to the curve of function $S_1(x_1)$. Hence, the optimal size for workers in the colony x_1 is determined only from $S_1(x_1)$, the task performance as a function of worker size. In the same manner, the optimal size of foraging workers x_2 is the value of the tangential point when we draw a line passing through the origin to the

curve of function $S_2(x_2)$. Hence, it is calculated only from $S_2(x_2)$, the foraging performance as a function of forager size.

As the sizes of two specialized workers are determined, the remaining variables are n_1 and n_2. We have two constraints: Equations (A.2) and (3). We note that the amounts of resources allocated to producing new workers in castes 1 and 2 are $n_1 x_1$ and $n_2 x_2$. Equation (A.2) is rewritten as follows:

$$\frac{n_1 x_1}{\frac{x_1}{S_1(x_1)C_1}} = \frac{n_2 x_2}{\frac{x_2}{S_2(x_2)C_2}},$$

which indicates that the ratio of resources used to produce the two castes is $\frac{x_1}{S_1(x_1)C_1}$ to $\frac{x_2}{S_2(x_2)C_2}$. In contrast, equation (3) indicates that their sum is equal to R. Hence, we have the following result:

$$n_1 = \frac{R}{x_1} \frac{\frac{x_1}{S_1(x_1)C_1}}{\frac{x_1}{S_1(x_1)C_1} + \frac{x_2}{S_2(x_2)C_2}}, \qquad \text{(A.7a)}$$

$$n_2 = \frac{R}{x_2} \frac{\frac{x_2}{S_2(x_2)C_2}}{\frac{x_1}{S_1(x_1)C_1} + \frac{x_2}{S_2(x_2)C_2}}. \qquad \text{(A.7b)}$$

which is the number of workers produced for the two different castes.

Supplementary Information

Appendix B

The number and the size of workers and switching age when all of them perform both tasks based on age

We consider the following:

$$\phi = \min[n_3 S_1(x_3)A_1(a_s), n_3 S_2(x_3)A_2(a_s)] \rightarrow \text{maximum}, \tag{B.1}$$

where $R = n_3 x_3$. Then, in a similar manner as that in Appendix A, we have the optimization of $n_3 S_1(x_3)A_1(a_s)$ under two constraints $n_3 S_1(x_3)A_1(a_s) = n_3 S_2(x_3)A_2(a_s)$ and $R = n_3 x_3$. By introducing Lagrange multipliers λ and μ, we have

$$\Phi = n_3 S_1(x_3)A_1(a_s) + \lambda\{n_3 S_1(x_3)A_1(a_s) - n_3 S_2(x_3)A_2(a_s)\} + \mu\{R - n_3 x_3\}. \tag{B.2}$$

We then choose n_3, x_3, and a_s in order to maximize Φ:

$$0 = (1+\lambda)S_1(x_3)A_1(a_s) - \lambda S_2(x_3)A_2(a_s) - \mu x_3, \tag{B.3a}$$

$$0 = (1+\lambda)S_1'(x_3)A_1(a_s) - \lambda S_2'(x_3)A_2(a_s) - \mu, \tag{B.3b}$$

$$0 = (1+\lambda)S_1(x_3)A_1'(a_s) - \lambda S_2(x_3)A_2'(a_s), \tag{B.3c}$$

and in addition, we have

$$S_1(x_3)A_1(a_s) = S_2(x_3)A_2(a_s). \tag{B.3d}$$

By eliminating μ from equations (B.3a) and (B.3b), we have

$$\frac{1+\lambda}{\lambda}\left\{\frac{S_1(x_3)}{x_3} - S_1'(x_3)\right\}A_1(a_s) = \left\{\frac{S_2(x_3)}{x_3} - S_2'(x_3)\right\}A_2(a_s). \tag{B.4}$$

By eliminating λ from equation (B.4) and equation (B.3c), we have

$$\frac{S_1(x_3)}{\left(\frac{S_1(x_3)}{x_3}-S_1{}'(x_3)\right)}\frac{A_1{}'(a_s)}{A_1(a_s)}=\frac{S_2(x_3)}{\left(\frac{S_2(x_3)}{x_3}-S_2{}'(x_3)\right)}\frac{A_2{}'(a_s)}{A_2(a_s)}. \qquad \text{(B.5)}$$

In the following, we calculate x_3 and a_s from equation (B.5) and equation (B.3d). Then, the number is obtained as $n_3 = R/x_3$.

Equation (B.5a) becomes

$$\frac{\frac{d}{da_s}\ln A_1(a_s)}{\frac{1}{x_3}-\frac{d}{dx_3}\ln S_1(x_3)}=\frac{\frac{d}{da_s}\ln A_2(a_s)}{\frac{1}{x_3}-\frac{d}{dx_3}\ln S_2(x_3)}, \qquad \text{(B.6)}$$

let $S_1(x) = \alpha_1 e^{-\beta_1/x}$ and $S_2(x) = \alpha_2 e^{-\beta_2/x}$. Noting

$$\frac{d}{da_s}\ln A_1(a_s) = \frac{d}{da_s}\ln\frac{1-\exp[-ua_s]}{u} = \frac{u\exp[-ua_s]}{1-\exp[-ua_s]} = \frac{u}{\exp[ua_s]-1},$$

$$\frac{d}{da_s}\ln A_2(a_s) = \frac{d}{da_s}\ln\left\{e^{-ua_s}\frac{1-\exp[-(u+v)(a_{max}-a_s)]}{u+v}\right\},$$

$$= -u - \frac{(u+v)\exp[-(u+v)(a_{max}-a_s)]}{1-\exp[-(u+v)(a_{max}-a_s)]} = -u - \frac{u+v}{\exp[(u+v)(a_{max}-a_s)]-1},$$

Equation (B.6) becomes

$$\frac{\beta_2-x_3}{x_3-\beta_1} = \frac{u+\frac{u+v}{\exp[(u+v)(a_{max}-a_s)]-1}}{\frac{u}{\exp[ua_s]-1}}. \qquad \text{(B.7a)}$$

Noting that the right-hand side is positive, the left-hand side is also positive, implying that x_3 is between β_1 and β_2. Suppose the optimal size of foragers β_2 is larger than that of within-colony workers β_1, then the optimal size of individuals who switch tasks from one to the other must have a body size between the two: $\beta_1 < x_3 < \beta_2$.

Thus, equation (B.5b) becomes

$$\exp[(\beta_2-\beta_1)/x_3]\frac{\alpha_1(u+v)}{\alpha_2 u} = \frac{1-\exp[-(u+v)(a_{max}-a_s)]}{\exp[ua_s]-1}. \qquad \text{(B.7b)}$$

A solution for equations (B.7a) and (B.7b) jointly gives the optimal choice of switching age a_s and the size of workers x_3.

The right-hand side (RHS) of equation (B.7a) is an increasing function of a_s. As a_s increases from 0 to a_{max}, the RHS increases from 0 to $+\infty$. The left-hand side (LHS) of equation (B.7a) is a decreasing function of x_3. As x_3 increases from β_1 to β_2, the LHS increases from 0 to $+\infty$. Combining these together, as a_s increases from 0 to a_{max}, x_3 decreases monotonically from β_2 to β_1. From this, we conclude that, on the $a_s - x_3$ plane, equation (B.7b) gives a curve with a negative slope connecting $(a_s, x_3) = (0, \beta_2)$ to $(a_s, x_3) = (a_{max}, \beta_1)$.

In contrast, the RHS of equation (B.7b) is a decreasing function of a_s. As a_s increases from 0 to a_{max}, the RHS monotonically decreases from $+\infty$ to 0. Let $a_s = \hat{a}_s$ be the value at which the RHS is equal to $\frac{\alpha_1(u+v)}{\alpha_2 u}$:

$$\frac{\alpha_1(u+v)}{\alpha_2 u} = \frac{1-\exp[-(u+v)(a_{max}-\hat{a}_s)]}{\exp[u\hat{a}_s]-1}.$$

The LHS of equation (B.7b) is a decreasing function of x_3. As x_3 increases from 0 to $+\infty$, it changes $+\infty$ to $\frac{\alpha_1(u+v)}{\alpha_2 u}$. Combining these, equation (B.7b) indicates that as a_s increases from 0 to $\hat{a}_s$, x_3 monotonically increases from 0 and diverges to $+\infty$. On the $a_s - x_3$ plane, equation (B.7b) shows a curve with a positive slope starting from $(a_s, x_3) = (0,0)$ and diverging at $a_s = \hat{a}_s$. For $\hat{a}_s < a_s < a_{max}$, x_3 is negative.

Hence, there is one intersection between the two curves, which is a unique solution of equations (B.7a) and (B.7b), and it satisfies $0 < a_s < \hat{a}_s$ and $\beta_1 < x_3 < \beta_2$. The number of workers is determined by $n_3 = R/x_3$.

Supplementary Information

Appendix C

Optimal strategy for exponentially growing colony

Consider a colony in which all of the resources produced by the activity of workers should be used for new worker production. The population is no longer in a stationary state. Instead, it grows exponentially with time. All of the variables related to the number of individuals become functions of time t, which is a parameter separate from age a. Below, we consider the task allocation pattern that realizes the fastest rate of colony growth.

Two castes of specialized workers

We should change equation (2) as follows:

$$R(t) = \min[I(t), F(t)], \tag{C.1a}$$

which indicates that all of the resources produced by the colony should be used for producing new workers. The performances of two tasks are as follows:

$$I(t) = \int_0^{a_{max}} S_1(x_1) f_1(a,t) da, \tag{C.1b}$$

$$F(t) = \int_0^{a_{max}} S_2(x_2) f_2(a,t) da. \tag{C.1c}$$

$f_i(a,t)$ is the number of workers of type i of age a at time t. These satisfy the following equations:

$$\frac{\partial f_1}{\partial t}(a,t) = -\frac{\partial f_1}{\partial a}(a,t) - u f_1 , \tag{C.2a}$$

$$\frac{\partial f_2}{\partial t}(a,t) = -\frac{\partial f_2}{\partial a}(a,t) - (u+v) f_2. \tag{C.2b}$$

The boundary conditions are as follows:

$$f_1(0,t) = n_1(t) \quad \text{and} \quad f_2(0,t) = n_2(t), \tag{C.3a}$$

$$R(t) = n_1(t)x_1 + n_2(t)x_2. \tag{C.3b}$$

This is a linear dynamical system; hence, it eventually converges to the stable age distribution, with all of the age classes growing exponentially with time. Let the exponential rate of growth be r. Then, all the functions above are proportional to e^{rt}. We replace each variable by the product of a quantity without time t multiplied by e^{rt}. For example, $f_1(a,t)$ is replaced by $\hat{f}_1(a)e^{rt}$, and $n_1(t)$ is replaced by $\hat{n}_1 e^{rt}$, and so on. We can rewrite these as follows:

$$\hat{R}e^{rt} = \min\left[\hat{I}e^{rt}, \hat{F}e^{rt}\right], \tag{C.4a}$$

$$\hat{I}e^{rt} = \int_0^{a_{max}} S_1(x_1)\hat{f}_1(a)e^{rt}da, \tag{C.4b}$$

$$\hat{F}e^{rt} = \int_0^{a_{max}} S_2(x_2)\hat{f}_2(a)e^{rt}da, \tag{C.4c}$$

$$r\hat{f}_1(a)e^{rt} = -\frac{d\hat{f}_1(a)}{da}e^{rt} - u\hat{f}_1(a)e^{rt}, \tag{C.4d}$$

$$r\hat{f}_2(a)e^{rt} = -\frac{d\hat{f}_2(a)}{da}e^{rt} - (u+v)\hat{f}_2(a)e^{rt}, \tag{C.4e}$$

$$\hat{f}_1(0)e^{rt} = \hat{n}_1 e^{rt} \quad \text{and} \quad \hat{f}_2(0)e^{rt} = \hat{n}_2 e^{rt}, \tag{C.4f}$$

$$\hat{R}e^{rt} = \hat{n}_1 e^{rt}x_1 + \hat{n}_2 e^{rt}x_2. \tag{C.4g}$$

Note that if all the quantities with this are multiplied by the same factor, the equations still hold. This implies that we can specify their ratio, but not their absolute values. It is plausible that we should search for the eigenfunction corresponding to the dominant eigenvalue r.

From equations (C.4d) and (C.4e), we have

$$\frac{d\hat{f}_1(a)}{da} = -(u+r)\hat{f}_1(a) \text{ and } \frac{d\hat{f}_2(a)}{da} = -(u+v+r)\hat{f}_2(a). \tag{C.5}$$

Together with (C.4f), we have

$$\hat{f}_1(a) = \hat{n}_1 \exp[-(u+r)a] \text{ and } \hat{f}_2(a) = \hat{n}_2 \exp[-(u+v+r)a]. \tag{C.6}$$

Using equations (C.4b) and (C.4c), we have

$$\hat{I} = \int_0^{a_{max}} S_1(x_1)\hat{n}_1 \exp[-(u+r)a]da$$

$$= S_1(x_1)\hat{n}_1 \frac{1}{u+r}(1 - \exp[-(u+r)a_{max}]), \tag{C.7b}$$

$$\hat{F} = \int_0^{a_{\max}} S_2(x_2)\hat{n}_2 \exp[-(u+v+r)a]da$$

$$= S_2(x_2)\hat{n}_2 \frac{1}{u+v+r}(1 - \exp[-(u+v+r)a_{max}]). \tag{C.7b}$$

By introducing

$$C_1(r) = \frac{1}{u+r}(1 - \exp[-(u+r)a_{max}]), \tag{C.8a}$$

$$C_2(r) = \frac{1}{u+v+r}(1 - \exp[-(u+v+r)a_{max}]), \tag{C.8b}$$

we have

$$\hat{R} = \min\left[\hat{I}, \hat{F}\right] = \min[S_1(x_1)\hat{n}_1 C_1(r), S_2(x_2)\hat{n}_2 C_2(r)], \tag{C.9}$$

and

$$\hat{R} = \hat{n}_1 x_1 + \hat{n}_2 x_2. \tag{C.10}$$

Now, our problem is to search for the optimal combination of x_1, $\hat{n}_1$, x_2, and $\hat{n}_2$ that attains the maximum of r. We maximize r under the constraints:

$$S_1(x_1)\hat{n}_1 C_1(r) = S_2(x_2)\hat{n}_2 C_2(r), \tag{C.11a}$$

$$S_1(x_1)\hat{n}_1 C_1(r) = \hat{n}_1 x_1 + \hat{n}_2 x_2. \tag{C.11b}$$

Using the Lagrange multipliers, we define

$$\Phi = r + \lambda\{S_1(x_1)\hat{n}_1 C_1(r) - S_2(x_2)\hat{n}_2 C_2(r)\} + \mu\{S_1(x_1)\hat{n}_1 C_1(r) - \hat{n}_1 x_1 - \hat{n}_2 x_2\}. \quad \text{(C.12)}$$

We then calculate the following: $0 = \partial\Phi/\partial\hat{n}_1$, $0 = \partial\Phi/\partial x_1$, $0 = \partial\Phi/\partial\hat{n}_2$, and $0 = \partial\Phi/\partial x_2$, which leads to $S_1(x_1)/x_1 = S_1'(x_1)$ and $S_2(x_2)/x_2 = S_2'(x_2)$, which are exactly the same as equations (4a) and (4b). The body size of each caste should be determined only by the function of how the task performance increases with body size. Equation (C.11a) states that the ratio of resource allocation to the two castes ($\hat{n}_1 x_1$ to $\hat{n}_2 x_2$) is equal to that of $x_1/\left(S_1(x_1)C_1(r)\right)$ to $x_2/\left(S_2(x_2)C_2(r)\right)$. The interpretation is the same as that in the stationary colony, but C_i $(i = 1,2)$ now has the time-discounting factor, and mortality u is replaced by $u + r$. We cannot determine the absolute value of $\hat{n}_1$ and $\hat{n}_2$, as we explained earlier. The other constraint equation (C.11b) gives the sum of $\hat{n}_1 x_1$ and $\hat{n}_2 x_2$. Combining this with the ratio, we have

$$\hat{n}_1 x_1 = S_1(x_1)\hat{n}_1 \mathrm{C}_1(r)\frac{x_1/\left(S_1(x_1)C_1(r)\right)}{x_1/\left(S_1(x_1)C_1(r)\right) + x_2/\left(S_2(x_2)C_2(r)\right)},$$

which becomes

$$1 = x_1/\left(S_1(x_1)C_1(r)\right) + x_2/\left(S_2(x_2)C_2(r)\right). \quad \text{(C.13)}$$

From equation (C.13), we can determine r as a single real number. Using this, we can state that the ratio of $\hat{n}_1$ to $\hat{n}_2$ is equal to the ratio of $1/\left(S_1(x_1)C_1(r)\right)$ to $1/\left(S_2(x_2)C_2(r)\right)$.

Workers of a single phenotype engage in both tasks

For the case of a single morph with the temporal switching of two tasks, we can develop a similar analysis. We again consider the situation in which the whole colony grows exponentially with a fixed age structure. We search for the optimal combinations of x_3 and a_s that attain the maximum exponential rate of growth r. In a similar way of calculation to that in the last paragraph, we solve the optimization of the following:

$$\Phi = r + \lambda\{\hat{n}_3 S_1(x_3) A_1(a_s, r) - \hat{n}_3 S_2(x_3) A_2(a_s, r)\} + \mu\{\hat{n}_3 S_1(x_3) A_1(a_s, r) - \hat{n}_3 x_3\}, \quad \text{(C.14a)}$$

where

$$A_1(a_s, r) = \frac{1}{u+r}(1 - \exp[-(u+r)a_s]), \quad \text{(C.14b)}$$

$$A_2(a_s, r) = \exp[-(u+r)a_s]\frac{1}{u+v+r}(1 - \exp[-(u+v+r)(a_{max} - a_s)]). \quad \text{(C.14c)}$$

From $0 = \partial\Phi/\partial\hat{n}_3$, $0 = \partial\Phi/\partial x_3$, and $0 = \partial\Phi/\partial a_s$, we have

$$0 = (\lambda + \mu) S_1(x_3) A_1(a_s, r) - \lambda S_2(x_3) A_2(a_s, r) - \mu x_3, \quad \text{(C.15a)}$$

$$0 = (\lambda + \mu) S_1{}'(x_3) A_1(a_s, r) - \lambda S_2{}'(x_3) A_2(a_s, r) - \mu, \quad \text{(C.15b)}$$

$$0 = (\lambda + \mu) S_1(x_3) \frac{\partial A_1}{\partial a_s}(a_s, r) - \lambda S_2(x_3) \frac{\partial A_2}{\partial a_s}(a_s, r). \quad \text{(C.15c)}$$

From these three, we can derive

$$\frac{\frac{\partial}{\partial a_s}\ln A_1}{\frac{1}{x_3} - \frac{\partial}{\partial x_3}\ln S_1} = \frac{\frac{\partial}{\partial a_s}\ln A_2}{\frac{1}{x_3} - \frac{\partial}{\partial x_3}\ln S_2}. \quad \text{(C.16)}$$

This is the same as equation (B.6) in the stationary colony, except that A_1 and A_2 depend on the future discounting of r and the mortality u is replaced by $u + r$. We can certainly derive an equation similar to equation (B.7a).

One constraint is $S_1(x_3)A_1(a_s, r) = S_2(x_3)A_2(a_s, r)$, which gives

$$\exp[(\beta_2 - \beta_1)/x_3]\frac{\alpha_1(u+v+r)}{\alpha_2(u+r)} = \frac{1-\exp[-(u+v+r)(a_{max}-a_s)]}{\exp[(u+r)a_s]-1}, \quad \text{(C.17)}$$

which is the equation in which we replace u with $u + r$. The second constraint is $S_1(x_3)A_1(a_s, r) = x_3$. This equation specified the value of r, the exponential growth rate. Note that we cannot specify $\hat{n}_3$ because the solution remains valid if we double its value. However, it is clear that the behavior of the optimal exponentially growing population is qualitatively the same as that for the stationary population.
